# GUItars: A GUI Tool for Analysis of High-Throughput RNA Interference Screening Data

**DOI:** 10.1371/journal.pone.0049386

**Published:** 2012-11-20

**Authors:** Asli N. Goktug, Su Sien Ong, Taosheng Chen

**Affiliations:** Department of Chemical Biology and Therapeutics, St. Jude Children's Research Hospital, Memphis, Tennessee, United States of America; CSIR-Institute of Microbial Technology, India

## Abstract

**Background:**

High-throughput RNA interference (RNAi) screening has become a widely used approach to elucidating gene functions. However, analysis and annotation of large data sets generated from these screens has been a challenge for researchers without a programming background. Over the years, numerous data analysis methods were produced for plate quality control and hit selection and implemented by a few open-access software packages. Recently, strictly standardized mean difference (SSMD) has become a widely used method for RNAi screening analysis mainly due to its better control of false negative and false positive rates and its ability to quantify RNAi effects with a statistical basis. We have developed GUItars to enable researchers without a programming background to use SSMD as both a plate quality and a hit selection metric to analyze large data sets.

**Results:**

The software is accompanied by an intuitive graphical user interface for easy and rapid analysis workflow. SSMD analysis methods have been provided to the users along with traditionally-used z-score, normalized percent activity, and *t*-test methods for hit selection. GUItars is capable of analyzing large-scale data sets from screens with or without replicates. The software is designed to automatically generate and save numerous graphical outputs known to be among the most informative high-throughput data visualization tools capturing plate-wise and screen-wise performances. Graphical outputs are also written in HTML format for easy access, and a comprehensive summary of screening results is written into tab-delimited output files.

**Conclusion:**

With GUItars, we demonstrated robust SSMD-based analysis workflow on a 3840-gene small interfering RNA (siRNA) library and identified 200 siRNAs that increased and 150 siRNAs that decreased the assay activities with moderate to stronger effects. GUItars enables rapid analysis and illustration of data from large- or small-scale RNAi screens using SSMD and other traditional analysis methods. The software is freely available at http://sourceforge.net/projects/guitars/.

## Background

High-throughput RNA interference (RNAi) screening has gained popularity in recent years as an efficient approach to elucidating gene functions. The availability of small interfering RNA (siRNA) and short hairpin RNA (shRNA) libraries that target the entire genome, the relative ease of use, and the efficiency of gene knockdown allows this reverse genetic approach to be amenable in a high-throughput manner [1]. When coupled with the use of small molecules, RNAi screening allows for the development of powerful chemical genetics-based synthetic lethality screens. However, the analysis and subsequent interpretation of large data sets obtained from genome-wide RNAi screens remains a tedious and slow process for researchers. Therefore, researchers who do not have the required resources to develop an in-house data analysis pipeline that meets their specific needs or to obtain commercial data analysis software heavily depend on open-access software packages available to them. One potential issue with these open-access packages is that some require substantial programming experience, which prevents researchers with limited programming skills to benefit from these resources. Additionally, currently available open-access high-throughput data analysis software uses the most common statistical methods that have been developed for the analysis of small molecule and RNAi screens, including mean difference, percent activity, z-score, and *t*-test statistics for normalization and hit selection purposes [2]–[9]. In recent years, a new statistical parameter, strictly standardized mean difference (SSMD), proposed by Zhang [10], [11] has become a widely used criterion for both screen quality control (QC) and hit selection, because the previously mentioned methods are associated with certain statistical drawbacks for the analysis of high-throughput screening data. Unlike these measures, SSMD addresses the magnitude of the RNAi effect and is more robust to sample size, which leads to comparable values across screens [12], [13]. By eliminating the effect of sample size and improving the control of false hit rates, SSMD was proved to be a more reliable parameter to be used in the analysis of high-throughput RNAi screens than the previously mentioned parameters [14]–[16].

To our knowledge, there has been no user-friendly open-access software package available to researchers that implements the SSMD algorithm for hit selection in high-throughput RNAi screening analysis. As such, one major advantage of GUItars over other tools (*e.g.* cellHTS2 [17], [18], RNAither [19]) is that it provides an SSMD-based high-throughput analysis tool for researchers working on RNAi screens. The traditional parameters for hit selection, including percent activity, z-score, and *t*-test are also available in our software package for the users who wish to compare their SSMD-based results with the traditional methods. With its automatic workflow, GUItars aims to facilitate the data analysis and visualization process for high-throughput RNAi screens. Notably, its user-friendly design enables researchers with little or no programming knowledge to set up the analysis via its graphical user interface (GUI) and carry out the entire calculation and visualization process by a single button click.

## Implementation

**Figure 1:**
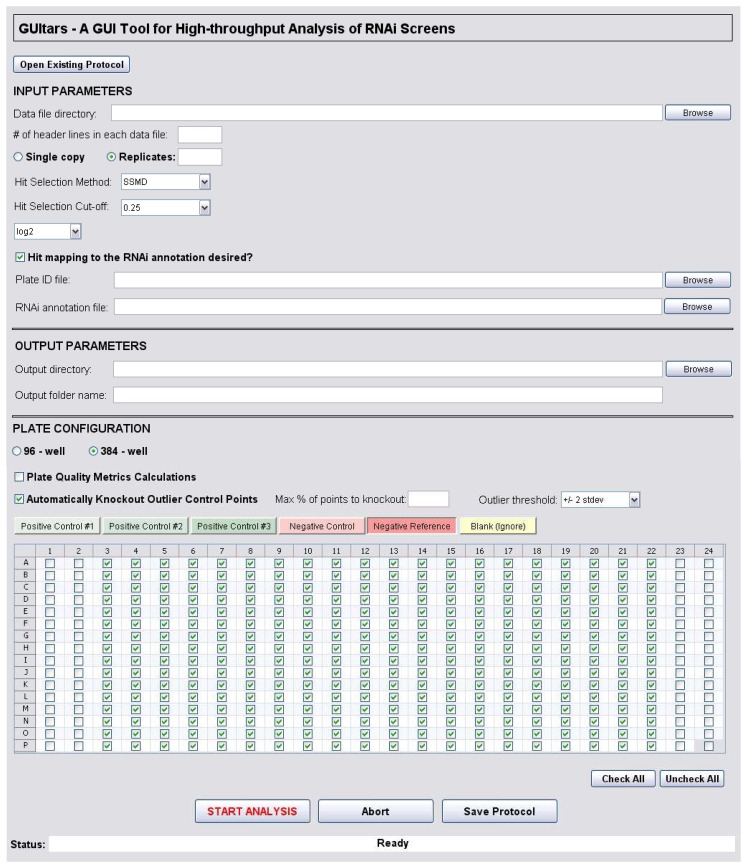
GUItars user interface. Graphical user interface of the standalone executable consists of three major sections: Input Parameters, Output Parameters, and Plate Configuration. Push buttons, pop-up menus, and checkboxes provide a user-friendly tool to easily enter the parameters and automatically carry out the entire analysis workflow with a single button click.

GUItars was programmed in MATLAB and made publicly available at (http://sourceforge.net/projects/guitars/) for researchers to analyze data from primary and confirmatory RNAi screens, performed either with or without replicates. It is a standalone executable, and no licensure is required. A signal intensity data file directory, a plate ID list (optional), and an RNAi annotation file (optional) need to be provided to run the software. The GUI for GUItars is designed for the end-user to easily enter the file destinations via popup dialog boxes. Alternatively, users can simply enter character strings containing the file paths in the corresponding fields. The GUI window enables the user to enter additional information such as screening method details (*e.g.*, single vs. replicate, 96 vs. 384-well), hit criterion, and plate configuration to carry out a smooth analysis. The user-modifiable fields on the GUI window are grouped into 3 major sections: Input Parameters, Output Parameters, and Plate Configuration (Figure 1).

### Input Parameters

**Figure 2:**
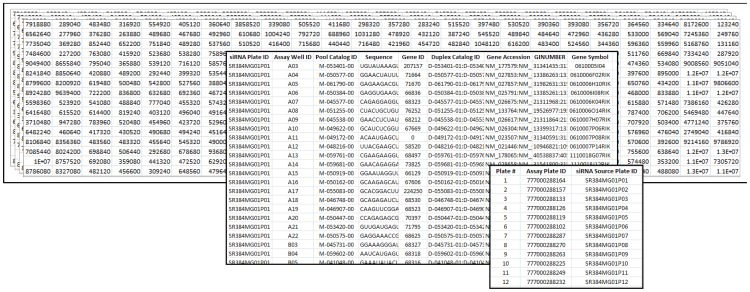
User input files required by GUItars. Three separate input files are required by GUItars: A “data file directory” containing individual files for each plate, an “annotation file” with first two columns containing RNAi source plate ID and assay plate well ID with a single header line, and a “plate ID file” with a single header line. An “annotation file” and a “plate ID” file are mandatory only if the “hit mapping to the RNAi annotation file” option is checked.

The “data file directory” contains the readouts from each assay plate, with each plate data point saved in a separate file (accepted file formats are tsv, tab-delimited txt for all operating systems; and additionally, csv, xls and xlsx for Windows users) (Figure 2). Instead of entering the well coordinates and the intensity values as a list, data must be provided in a matrix form in 16-by-24 or 8-by-12 well format for 384- or 96-well plate screens, respectively. The well coordinates are automatically captured, and the well IDs are assigned accordingly. Number of header lines, which should be identical for each data file, must be specified in the “# of header lines in each data file” field on the GUI window. The processed data content is summarized in the “Raw_data_compiled.tsv” file output. Since GUItars is capable of running analysis for screens with or without replicates, the user must define the screening method of interest by selecting either the “single copy” or “replicates” radio button. If there are replicate assay plates with the same RNAi content, the “replicates” option should be selected, and the number of replicates must be specified. An exception to this rule is if the user prefers to evaluate each assay plate independently, keeping in mind that a different SSMD calculation will be performed. For screens with replicates (interplate), the number of assay plate replicates corresponding to each RNAi source plate has to be equal; otherwise, each condition should be analyzed separately.

The “hit selection method” drop-down menu contains a list of available analysis methods with GUItars. For user convenience, the list contents and the associated parameter options are interactively updated and become visible for further analysis specifications. For screens without replicates, the list includes percent activity, z-score, robust z-score, SSMD, and robust SSMD options. For screens with replicates (3 or more), the user can choose between *t*-test and SSMD. The “hit selection cut-off” field is provided for user-defined threshold selection, and the value to be entered should be a reasonable cut-off value for the analysis method that will be used. To guide the user through the SSMD cutoff selection, predefined cutoff values are provided in a drop-down menu based on criteria developed by Zhang [11]. With methods other than percent activity and *t*-test, the user-defined “hit selection cut-off” is applied to automatically identify hits that either increase or decrease assay signals, corresponding with assay output readings above the (+) cutoff and below the (−) cutoff, respectively. Another drop-down list is also provided with options to perform the analysis either on raw or log-transformed (log_2_ or log_10_) data.

“Hit mapping to the RNAi annotations” is an optional feature that allows the users to choose whether or not matching the assay plate (hit) wells to the RNAi annotations is desired. The following fields will be enabled or disabled based on the user's selection: “plate ID file” and “RNAi annotation file”. The “plate ID file” contains the assay and RNAi source plate ID information in one of the accepted file formats as mentioned above (Figure 2). For accurate mapping purposes, the RNAi source plate IDs must match the plate IDs in the first column of the “RNAi annotation file”. If the hit mapping option is selected, the “plate ID file” will be used as the master guide for data import. The file names within the data file directory must contain the unique assay plate IDs defined in the plate ID file. If the plate ID field is left empty for either the assay or RNAi source plate, it will be replaced with “no plateID” notation, and the rest of the analysis and mapping will be affected. In screens with replicates, hit mapping is required, and the assay plate IDs for the replicate plates must be entered consecutively in the “plate ID file”.

The “RNAi annotation file” lists the RNAi source plate contents/annotations containing a header line in the first line (Figure 2). It is mandatory that the first and second columns of the file contain RNAi source plate IDs and the corresponding assay well IDs, respectively. The rest of the columns can be as many as desired comprising any relevant gene information (*e.g.*, gene ID, accession number).

### Output Parameters

In addition to an Excel file generated with multiple tabs containing comprehensive analysis results (Windows only), output data are also written into individual tab-delimited files for user convenience (all operating systems). All the graphical outputs are stored as JPEG images and written in HTML format, which can be viewed on any web browser. The images are also saved in MATLAB figure format (.fig) which can be printed or saved in higher resolution in various formats such as tif, png, eps, and pdf, as needed. A separate GUI tool to open the MATLAB figures is included in the software package available at the project home page (http://sourceforge.net/projects/guitars/) for users without licensed MATLAB software.

A desired directory and a folder name (maximum 25 characters) to save all the output files can be specified in the “output directory” and “output folder name” fields, respectively. The output folder name will be further customized automatically with date and time information to avoid overwriting existing files.

### Plate Configuration

For ease of use, well configurations are defined on an interactive plate map provided in the GUI window. In this section, the plate format and the well coordinates should be assigned using the corresponding radio buttons and tabs above the plate configuration panel. The screening plate format can be either in 96-well or 384-well layout, which must match the data format in the “data file”. Based on the user's selection, the plate configuration panel is dynamically updated to the selected plate format. If the “plate quality metrics calculations” option is checked, negative controls and at least one of the positive control well positions must be specified in the plate map.

The user can specify the well positions on the plate map by checking the boxes corresponding to each well and can navigate through different well types using the colored tabs. GUItars is capable of handling three sets of positive controls in plate QC calculations. To avoid conflicts arising from selecting the same well in more than one tab, the selected wells in the active tab will automatically be disabled in the inactive tabs, and the user will be prompted with a warning message when attempting to check a disabled well position, except in certain circumstances (*e.g.*, a negative control well can also be defined as a negative reference well). For ease of use, the “Check All” and “Uncheck All” buttons can be used to perform these actions simultaneously on all wells within the active tab.

As a side note, for a screen in which a high hit rate is not necessarily expected (i.e., a screen not using a focused library), all wells containing the RNAi samples should be designated as negative references. On the other hand, negative controls can be used as negative reference if a focused library or a confirmatory screen is being used as described by Zhang [13]. In GUItars, negative reference wells are used as a primary data source in scoring calculations.

Wells that do not contain any controls or RNAi samples should be designated as blank, so they will then be excluded from the calculations for all plates. If only a subset of the plates contain blank wells, or if certain outliers are desired to be manually excluded from the analysis at plate level, then the data points corresponding to those wells should be replaced with the “NaN” notation in the individual data files. GUItars also includes an algorithm to automatically knock out the outlying data points from the control wells based on user demand. For that, the user should check the “automatically knockout outlier control points” option and fill out the corresponding enabled fields.

The current analysis session can be saved and retrieved via the “Save Protocol” and “Open Existing Protocol” buttons, allowing the users to reuse or share their analysis setup with others. Once all the required fields are completed in the GUI window, analysis can be started by clicking the “Start Analysis” button, and the status of the process can be monitored via the status bar. When the analysis is started, preprocessing of the user input files is performed, and the data sets are checked for completeness. If any unexpected data formatting or analysis issues are encountered, the user is informed by a pop-up warning message, and the program is aborted. GUItars is developed with a robust error capturing mechanism against operator errors with 20 various warning messages to pinpoint the problem and provide an easy-to-use tool.

## Results and Discussion

**Figure 3:**
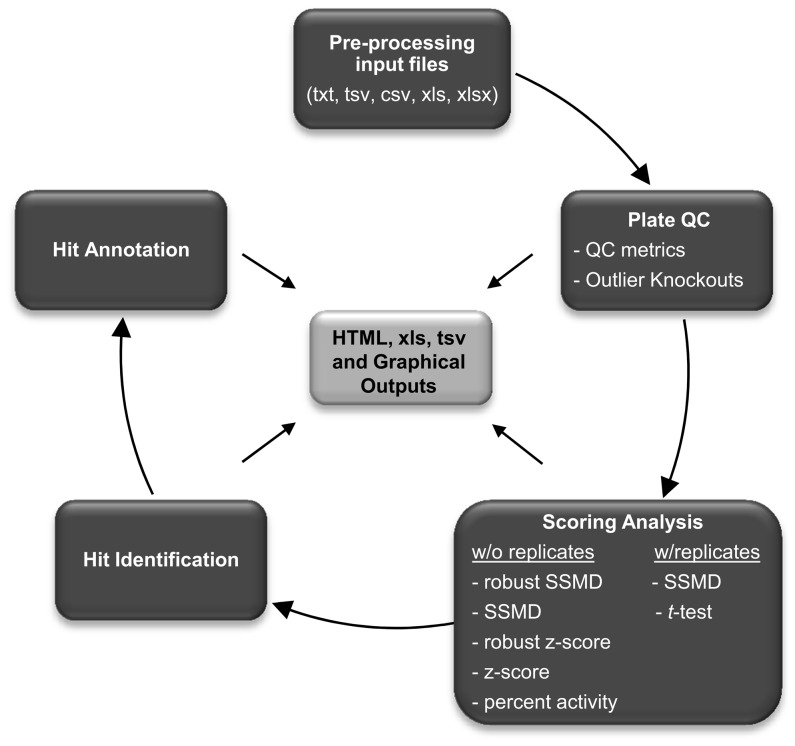
General workflow of high-throughput data analysis with GUItars.

Data analysis is performed and visualized according to the workflow presented in Figure 3. Data sets are processed in three major steps, as follows: Plate QC, scoring and hit selection, and hit annotation. We demonstrated the analysis process and graphical outputs using a 12-plate siRNA (3840-gene pooled mouse siRNA library) screen without replicates in a 384-well format with a luminescence-based assay readout with an emphasis in the importance of distinct visualization approaches, which are chosen to be implemented in GUItars as default. In the demonstrated data set, 320 sample wells were used as negative reference in each plate. For plate QC calculations, 16 different wells were defined as negative control, positive #1 and #2, and 8 wells were defined as positive control #3.

### Plate QC

**Figure 4:**
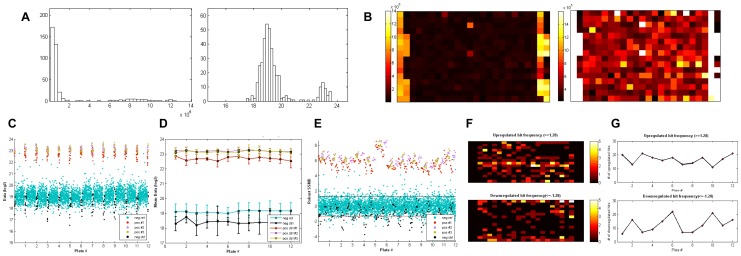
Graphical outputs demonstrated on a 12-plate siRNA screen analyzed with the robust SSMD method with GUItars. (A) Raw data (left) and log_2_-transformed data (right) histograms of each plate showing the original data distribution and effect of data transformation (one representative plate is shown). (B) Original scale (left) and rescaled (right) heat maps of each plate helping to capture systematic errors (one representative plate is shown). (C) Column-wise plate-series plot. (D) Screen-wise line plot for average control readings showing a clear separation between negative control and positive controls that is consistent throughout the screen. (E) Screen-wise SSMD score scatter plots with cutoff lines at 1.28 and −1.28 for signal-increasing and signal-decreasing hits, respectively. (F) Hit distribution heat maps for signal-increasing (top) and signal-decreasing (bottom) hits. (G) Screen-wise hit counts for signal-increasing (top) and signal-decreasing (bottom) hits.

As a common practice in RNAi screens, logarithmic transformation of the raw data is often performed to achieve a symmetrically distributed data set by normalizing highly skewed distributions [16], [20]. If the user chooses to log-transform the raw data, data distribution histograms of each plate are generated for raw data as well as the transformed data, as seen in Figure 4A. With the aid of histograms, one can decide whether the applied transformation method has met the data distribution expectations and whether the hits identified from any of the plates should be evaluated with skepticism.

Plate heat maps are provided to help with the inspection of systematic errors, which may commonly include patterns introduced by liquid handling instruments or edge effects associated with incubation quality (Figure 4B). However, in assays with a high signal window, one can misleadingly conclude an inexistence of systematic errors because the color scaling is biased. Therefore, a second set of heat maps is provided with a color scaling ranging between minimum(mean of negative reference)−2*minimum(standard deviation (SD) of negative reference) and maximum(mean of negative reference)+2*maximum(SD of negative reference). As seen in Figure 4B, it is easier to observe systematic patterns in the heat map on the right than in the one on the left, although there are no evident errors observed in the screen shown.

While a heat map is a good tool for visually identifying systematic errors at the individual plate level, a plate-series plot is also an expedient way to review the overall performance of the screen at a glance. With GUItars, plate-series plots are generated in row-wise and column-wise formats as so that the row and column effects can be captured easily. A representative column-wise plot is shown in Figure 4C, and it is confirmed that there are no major systematic errors.

**Figure 5:**
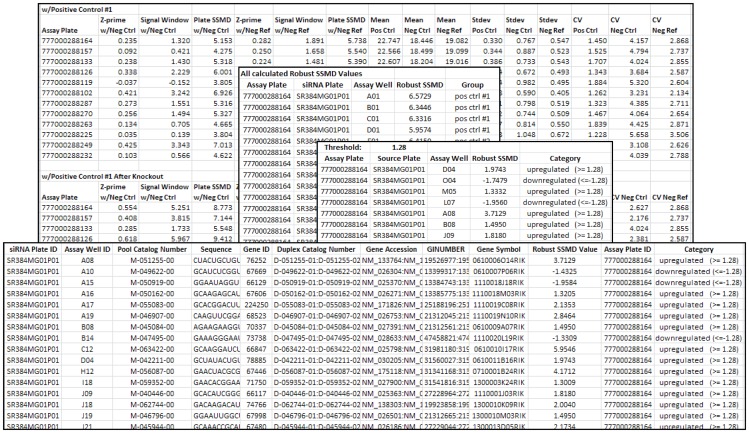
Excel readable output file. Individual tab-delimited output files as well as a comprehensive Excel file are generated with the following information: Plate QC calculations before and after control outlier knockout, scores for all wells classified by well type, scores for hit wells classified by hit type (i.e., signal-increasing or signal-decreasing), and annotated hit list (optional) with corresponding scores.

With GUItars, calculations of the comprehensive plate QC parameters are performed based on the user's positive control, negative control, and negative reference well designations (after data transformation, if selected) when the “plate quality metrics” option is checked. Although RNAi screens share certain similarities with small molecule screens, there are many aspects in which they differ. Notably, the Z′-factor [21], a measure of the quality of a screen, is considered acceptable for small molecule screens when it is greater than 0.5. However, for RNAi screens, Z′ is usually less than 0.5. As such, using the Z′-factor, RNAi screens are usually less robust than small molecule screens. As proposed by Zhang [10], SSMD can be a better alternative plate QC metric than the Z′-factor. Zhang has shown that, as a quality measurement in high-throughput assays, SSMD is based on a firm statistical theory and clear probabilistic meaning, as opposed to the Z′-factor, which lacks a statistical basis and entails a relatively arbitrarily chosen pass/fail cutoff. Moreover, Zhang has illustrated that quality assessment by the Z′-factor may be misleading for screens with moderate or strong positive controls, while the SSMD-based QC metric can quantify plate quality for screens with positive controls with various activities [22]. Although the Z′-factor is applicable to assays with single readout, another version of the formula which can incorporate multiple readouts via linear projection of the data is developed by Kümmel et al. [23] for applications such as high content screening. However, it was not integrated into GUItars' plate quality calculations since multivariate applications are beyond the scope of this study. In GUItars, SSMD (method-of-moment estimate), as a primary plate QC metric, is calculated in addition to the Z′-factor and signal window for each positive control independently in a plate-wise manner. A verbose quality metrics summary is written to an output file, which also includes mean, SD, and coefficient of variation values of positive control, negative control, and negative reference wells (Figure 5). SSMD, Z′-factor, and mean values of controls (Figure 4D) are also graphed on separate line plots for easy plate-to-plate comparison.

The outlier knockout is then performed if it is selected by the user in the analysis setup. The knockout algorithm is designed to disregard the wells whose values are greater or less than the mean ± x SD of all the wells in the particular control set, starting from the most outlier well, where x is the user-defined outlier threshold. The knockout process ends once the maximum number of points to knock out is reached or when there are no points left satisfying the above criteria, whichever occurs first. In the screen shown, we chose to knock out 30% of the points with values more extreme than ±2 SD. All the plate quality metrics and visualizations are regenerated after the control outliers are knocked out so that the user can see the comparable results of the process.

### Scoring and Hit Selection

GUItars is primarily designed to enhance and facilitate the application of the SSMD-based scoring and hit selection method on high-throughput RNAi screens. In addition to SSMD and robust SSMD, GUItars can apply other commonly used methods, including normalized percent activity, z-score, and robust z-score for screens without replicates and the paired *t*-test for screens with replicates, as summarized by Zhang [24]. Hence, the user has the flexibility to choose among various scoring methods from the user interface. The other methods mentioned above, which are incorporated into the currently available open-access software packages, however, are associated with certain statistical drawbacks for the analysis of high-throughput screening data. In a primary screen without replicates, the mean difference and percent activity measures fail to incorporate data variability in the hit selection [2]. On the other hand, the regular z-score and SSMD methods assume that the variability of a sample RNAi is same as the variability in a negative reference group [25]. It is known that the robust versions of the z-score and SSMD are less sensitive to outliers due to the replacement of mean and SD with median and median absolute deviation (MAD) [9], [26], [27]. Therefore, the use of the robust versions of these methods is recommended for high-throughput screen analysis. Although z-score and SSMD are linearly related parameters for screens without replicates, the primary difference arises when the SSMD is applied for screens with replicates, in which case z-score is not a valid criterion to score RNAi samples with various variability [13]. On the other hand, in confirmatory screens with replicates, *p*-values associated with a z-score or *t*-test determines whether the null hypothesis, which is the mean of an RNAi sample being equal to the mean of the negative reference group, is accepted or not. Also, sample size has major influence on the resulting *p*-values due to the formulation of these tests. In contrast, SSMD across replicates provides a direct means of measuring the RNAi effect compared with the negative reference wells without the effect of sample size. In GUItars, we use the uniformly minimal variance unbiased estimate (UMVUE) of SSMD in the sample scoring [11].

In the siRNA screen shown, we applied robust SSMD hit selection at a cutoff of 1.28 on log_2_-transformed data. As a result of the analysis, associated scores for all wells in every plate were written into an output file with the corresponding assay plate ID, RNAi plate ID, and assay plate well ID, which is categorized based on the well types (i.e., positive control, negative control, and negative reference) (Figure 5). To visualize the scores and the hit distributions in each plate, individual scatter plots are generated, in which horizontal lines are drawn corresponding to the cutoff used to select hits that either increase or decrease the assay activities. A series plot showing the well scores of all the screened plates is also necessary to explore the plate-to-plate variance within the screen. As seen in a representative graph in Figure 4E, the SSMD values and hit distributions are consistent among all the plates, except in plate 6, which has smaller plate median and MAD values, yielding higher SSMD scores.

In GUItars, the positive control and the negative reference wells are used as the high and low signals in the normalized percent activity calculations whereas only the negative reference wells are used in all the other methods. When the percent activity method is applied, the user has to define which control corresponds to the high signal and whether the hits are selected above or below the selected cutoff. If the *t*-test method is chosen, the “hit selection cut-off” is treated as the maximum *p*-value below which the hits are considered significant. Unlike SSMD, by the nature of the *t*-test statistics, the program outputs cannot classify whether an RNAi hit increases or decreases the assay signal.

The scoring calculations are followed by hit selection based on the cutoff defined by the user excluding the user-defined control and blank wells. The hit well IDs and the hit categories (i.e., increased or decreased signal) are determined above and below the positive and negative cutoff values (in percent activity and *t*-test, only the positive cutoff is considered), and written into an output file (Figure 5). A hit distribution heat map is utilized as a means to detect the effects of systematic errors in the final hit distribution. It is expected that the hits are distributed randomly among the wells in all plates; therefore, if a particular well is a “hotspot” for hits in most of the plates, that may be an indication of systematic errors. To provide a visual summary of the hit distribution, the frequency of a well being identified as a hit in all plates within a screen is calculated for each well and presented in separate heat maps for signal-increasing and signal-decreasing hits (Figure 4F). Additionally, the number of hits identified from each plate should also be consistent within a screen unless certain plates are specifically designed to lead to higher hit rates. Therefore, a screen-wise line plot of the number of signal-increasing and signal-decreasing hits is selected as another graphical output mode, allowing the user to inspect for the overall hit selection performance (Figure 4G). Thus, a hit distribution map along with a hit counts line plot provides crucial information for determining the reliability of the screen results.

### Hit Annotations

If the “hit mapping to the RNAi annotation” option is selected, a hit mapping algorithm is used to match the hit well IDs of the assay plates with the corresponding RNAi plate annotations according to the user input “plate ID” and “RNAi annotation” files. For screens with replicates, one should use extra care to input the same RNAi source plate ID for all corresponding copies of the assay plates. An output file containing the assay plate ID, score, and the category of the hit wells is generated with the matched RNAi source annotations (Figure 5).

## Comparisons

The two leading open access high-throughput analysis software packages for RNAi screens are cellHTS2 [17], [18] and RNAither [19] in the BioConductor package, and both are developed in the R programming language. Compared with both cellHTS2 and RNAither, the most important advantage of GUItars is the implementation of SSMD as both QC and hit selection metrics. While both of these tools offer high flexibility and powerful features for data normalization and annotation, their hit identification algorithms rely on frequently used techniques such as mean difference, percent activity, mean ± x SD, median ± x MAD, and *t*-test. For researchers who would like to have a hit identification strategy that has a statistical basis and a control on false hit rates, especially on screens with samples with extreme (large or moderate) effects, the SSMD technique is a more suitable option. However, as also stated by Birmingham et al. [28], it is not easy for nonprogrammers to carry out the calculations from scratch, and no open-access packages have implemented SSMD in their workflow yet. The user-friendly graphical interface of GUItars does not require any command entries from the end-user and makes the SSMD analyses achievable for researchers with no programming background. One of the major advantages of the SSMD-based hit selection metric over the other methods is the feasibility of ranking and classification of the RNAi hits by quantifying the size of RNAi effects. Therefore, GUItars not only selects the hits based on a user-defined hit cutoff, but also provides a list of gene counts based on their effect types (according to the thresholds introduced by Zhang [12]) to guide the researchers for more deliberated hit selection for validation studies. A table of classified siRNA counts from the screen shown is presented in Table 1.

**Table 1:** siRNA counts classified by effect sizes.

*Type*	*RNAi Effect Classes*	*RNAi Effect Cutoffs*	*Counts*
upregulated	≥5	extremely strong	2
upregulated	5>SSMD ≥3	very strong	15
upregulated	3>SSMD ≥2	strong	39
upregulated	2>SSMD ≥1.645	fairly strong	34
upregulated	1.645>SSMD ≥1.28	moderate	110
upregulated	1.28>SSMD ≥1	fairly moderate	169
upregulated	1>SSMD ≥0.75	fairly weak	235
upregulated	0.75>SSMD ≥0.5	weak	338
upregulated	0.5>SSMD ≥0.25	very weak	417
upregulated	0.25>SSMD ≥0	extremely weak	561
downregulated	0>SSMD ≥−0.25	extremely weak	542
downregulated	−0.25>SSMD ≥−0.5	very weak	481
downregulated	−0.5>SSMD ≥−0.75	weak	365
downregulated	−0.75>SSMD ≥−1	fairly weak	238
downregulated	−1>SSMD ≥−1.28	fairly moderate	144
downregulated	1.28>SSMD ≥−1.645	moderate	93
downregulated	−1.645>SSMD ≥−2	fairly strong	34
downregulated	−2<SSMD ≥−3	strong	21
downregulated	−3<SSMD ≥−5	very strong	2
downregulated	>−5	extremely strong	0
zero	= 0	no effect	0

GUItars output with gene counts ranked based upon the criteria presented by Zhang [12]. Data is generated from a 12-plate luminescence-based assay with 3840 total genes.

## Conclusions

A MATLAB-based open-access software tool, GUItars, for the analysis and illustration of RNAi screening data is described. The user-friendly graphical interface enables rapid analysis setup with the aid of specially designed pop-up menus, push buttons, and a panel of plate configuration checkboxes instead of requiring command entries from the end-user. GUItars uses the UMVUE estimate of SSMD formulas for hit selection, which is preferred for its ability to diminish sample size effects and the false hit rate, making it superior to other widely used high-throughput screening analysis methods. The program can handle the analysis for screens with or without replicates in 96- or 384-well formats. With the demand for higher throughput screening formats, the use of 1536-well plates is gaining a foothold. Therefore, future versions of this software capable of handling this assay format can be made available. For comparison purposes, non-SSMD-based methods such as percent activity, z-score, and *t*-test are also provided as scoring options. In addition to the tab-delimited and Excel file outputs, the graphical outputs generated with GUItars display the most relevant information that is extracted from the input data sets and the analysis results. The general workflow of the program is demonstrated using an siRNA screen with luminescence as the readout. The software features will be further improved by the addition of various data normalization options for edge effect and systematic error corrections [29]–[32]. Since GUItars can handle data input files in tsv, tab-delimited txt, csv, xls and xlsx fomats, it is capable to process the data directly exported from most microplate readers. Even though GUItars is designed for the analysis of RNAi screening data, it is also applicable for small molecule screens. In summary, this automated graphical analysis tool greatly reduces the time necessary to perform high-throughput screening analysis tasks manually, especially for SSMD-based analysis purposes.

## Availability and Requirements

- Project name: GUItars
- Project home page: http://sourceforge.net/projects/guitars/
- Operating system(s): Microsoft Windows, Linux and Mac versions are available
- Programming language: MATLAB
- Other requirements: Web browser
- License: no license needed
- Any restrictions to use by non-academics: none

The source code, standalone executables and the exemplary dataset are provided in individual zip folders for each operating system along with the complete set of analysis results of the demonstrated screen.
